# A Potential Ratio for Detecting Subclinical Atherosclerosis: Insight into Advanced NMR Lipid Profiles in Severe Obesity

**DOI:** 10.1007/s12265-025-10696-x

**Published:** 2025-10-29

**Authors:** Júlia Carmona-Maurici, Iratxe Eskubi-Turró, Anna Viñas, David Ricart-Jané, Mª Dolores López-Tejero, Núria Amigó, Marcelino Bermúdez, Juan Antonio Baena-Fustegueras, Julia Peinado-Onsurbe, Eva Pardina

**Affiliations:** 1 Departament de Bioquímica i Biomedicina Molecular, Facultat de Biologia, Universitat de Barcelona (UB), Diagonal 643, 08028 Barcelona, Spain; 2 Spanish Biomedical Research Centre in Diabetes and Associated Metabolic Disorders (CIBERDEM), Madrid, Spain; 3 Biosfer Teslab SL, Department of Basic Medical Sciences, Institut d’Investigació Sanitària Pere Virgili (IISPV), Universitat Rovira I Virgili (URV), Reus, Spain; 4 Vascular and Renal Translational Research Group, IRBLleida, Lleida, Spain; 5 Spanish Research Network for Renal Diseases (RedInRen, ISCIII), Lleida, Spain; 6 Endocrinology Surgery Unit, Arnau de Vilanova University Hospital (UdL), Lleida, Spain

**Keywords:** Lipoproteins, Atherosclerosis, Severe obesity, Bariatric surgery, Nuclear magnetic resonance

## Abstract

Individuals with severe obesity (SO) are at a high risk of developing cardiovascular disease, but traditional lipid parameters are insufficient for accurately assessing the risk. This study aims to investigate the advanced characteristics of lipoproteins that may contribute to subclinical atherosclerosis in SO and the impact of bariatric surgery (BS). The study included 37 patients with SO with a one-year follow-up post-BS, and 40 control subjects. Advanced lipoprotein profiles were assessed using nuclear magnetic resonance. BS normalized proatherogenic lipoprotein alterations in SO. Small LDL and medium HDL particle numbers differed between plaque and non-plaque groups. A ratio of these particles showed an AUC of 83%, suggesting it could effectively predict subclinical atherosclerosis. Advanced NMR analysis offers more specific information on lipid profiles in SO. The small LDL-P to medium HDL-P ratio could be a valuable tool for detecting and managing subclinical atherosclerosis in this population.

**Graphical Abstract:**

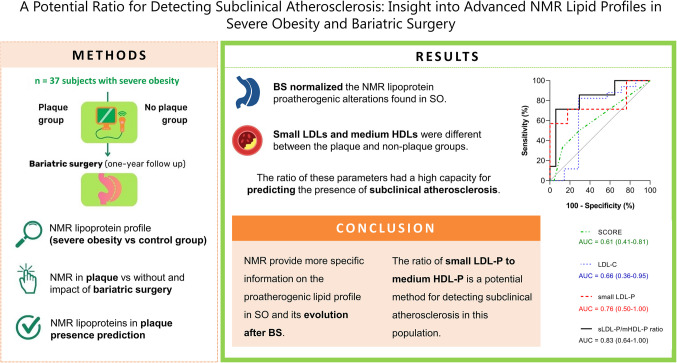

## Introduction

Cardiovascular diseases (CVDs) are the leading cause of mortality and disability in the world [1]. Dyslipidemia (DLP) is one of the most common risk factors for CVD and further heightens the risk of developing CVD when combined with severe obesity (SO) [2]. Obesity-associated DLP is characterized by an increased serum concentration of triacylglycerides (TGs) accompanied by a decreased level of high-density lipoprotein cholesterol (HDL-C). Meanwhile, in SO, low-density lipoprotein cholesterol (LDL-C) concentrations can be mildly increased or optimal [3].

The cardiovascular risk linked to DLP arises from its contribution to the development of atherosclerosis, the underlying pathological process in most CVDs. Atherosclerosis occurs through the accumulation of lipid deposits on the arterial walls that leads to the formation of atheromatous plaques during a silent phase known as subclinical atherosclerosis [4–7]. When a plaque ruptures, it causes acute events such as ischemic heart disease or strokes [1].

Arterial ultrasound is the recommended method for detecting subclinical atherosclerosis [8]. However, it is not available in all healthcare settings. Furthermore, the increased amount of subcutaneous and perivascular adipose tissue in people with SO complicates the detection of plaques [9].

Hence, the evaluation of lipid alterations is essential for assessing cardiovascular risk and have been used as modifiable and fundamental factors in prevention strategies in recent decades [10]. However, despite the progress achieved to date and an adequate control of the lipid parameters that are assessed in routine clinical practice, subjects with SO experience a high residual cardiovascular risk and a large proportion of subjects undergoing treatment may suffer cardiovascular events despite showing normal lipid levels and cardiovascular risk scores [11–13]. Notably, in a prior study, we reported no significant alterations in serum lipids (total cholesterol, LDL-C, and HDL-C) in subjects with obesity and atherosclerotic plaques [14]. Thus, conventional lipid parameters may underestimate the cardiovascular risk in subjects with SO.

Beyond the cholesterol content, other lipoprotein characteristics could have a role in the cardiovascular risk and atherogenicity [15]. Advanced lipoprotein testing with nuclear magnetic resonance (NMR) analysis offers information on particle number (P), size, diameter, and the internal content (esters of cholesterol and TG) of each particle, including very-low-density lipoprotein (VLDL), intermediate-density lipoprotein (IDL), LDL, and HDL [16]. A growing body of recent evidence supports the potential for using an advanced lipoprotein profile to assess cardiovascular risk [17].

Individuals with SO face a high cardiovascular risk, but traditional lipid parameters are insufficient for precisely assessing this risk. Thus, this study aimed to investigate the NMR characteristics of lipoproteins, including their number, content, and size, which may contribute to subclinical atherosclerosis in SO. Moreover, the impact of bariatric surgery (BS) on these lipoprotein features was studied.

## Subjects and Methods

The Hospital Ethics Committee approved the study protocol and all subjects provided written informed consent before study participation. All procedures performed in the study were conducted following the ethical standards of the national research committee and were in accordance with the Declaration of Helsinki.

### Study Population and Design

This prospective study included 37 subjects with SO (26 women and 11 men, mean age of 44 years) and 40 subjects without obesity (control group of 32 women and 8 men, mean age of 43 years), who were recruited at the Hospital Arnau de Vilanova (Lleida, Spain). Subjects in the control group did not have DLP and type 2 diabetes mellitus (T2D).

The presence of atheromatous plaques was determined by ultrasound, as described before [14], in the 37 subjects with SO. The 37 subjects had undergone laparoscopic BS, but only 24 had completed the one-year follow-up. Seven out of the 24 subjects who finished the study presented atheromatous plaque.

All subjects in the SO group met the necessary criteria for BS: a body mass index (BMI) ≥ 40 or ≥ 35 kg/m^2^ with at least one comorbidity, including hypertension (HT), T2D, DLP, obstructive sleep apnea, or weight-induced rheumatic disease diagnosed according to the National Cholesterol Education Program (NCEP) [18]. None of the subjects were diagnosed with inflammatory or infectious diseases and none were receiving anti-obesity or anti-inflammatory drugs at the time of the study. Subjects were excluded if they had any previous cardiovascular event, hypothyroidism, endocrine diseases other than T2D, or any neoplastic, renal, hepatic or active systemic disease.

To analyze lipoprotein profiles, plasma samples were collected under fasting conditions between 08:00 and 10:00 am. All measurements were performed before BS in the 37 subjects with SO and 12 months after surgery (12 M) in the 24 who had completed the follow-up. The control group was assessed at only one time-point.

The study population was characterized by analyzing the lipoprotein parameters that are assessed in routine clinical analytics to report the current practices employed in clinical practice to evaluate the cardiovascular risk, and the SCORE risk prediction algorithm to estimate 10-year risk of cardiovascular disease was calculated [19]. We then implemented a three-block study design to enhance the understanding of the cardiovascular risk assessment with lipoproteins.

First, we conducted a thorough investigation of the NMR lipoprotein profile in SO by comparing the lipoprotein parameters between the cohort of 37 subjects with SO and the control group. Then, in the 24 out of the 37 subjects who had completed the follow-up, we compared the NMR lipoprotein profiles between subjects with arterial plaque and those without, identifying any differential parameters and analyzing the impact of BS. Finally, we undertook an exploratory study on the capacity of differential NMR parameters to predict the presence of arterial plaques in SO.

### Lipoprotein Analysis by NMR Spectroscopy

Serum samples (250 μL) were analyzed in Biosfer Teslab (Reus, Spain) to determine the lipoprotein profile using the NMR-based Liposcale® test.

Two-dimensional diffusion NMR is based on the study of particle mobility within a fluid (serum or plasma) that is associated with particle size. This technique can directly measure the quantity, size, and composition of lipoprotein fractions and subfractions (large, medium, and small), as previously reported [20]. The concentration of each particle subfraction was calculated by dividing the lipid volume by the particle volume [21]. The weighted average of the VLDL, LDL, and HDL particle sizes was calculated [16].

### Statistical Analysis

Results are expressed as the mean ± SD. Normally distributed quantitative variables were assessed with the Shapiro–Wilk test. Statistical differences between subjects with and without plaques and their evolution with BS were assessed using two-way repeated measures ANOVA and the Bonferroni multiple comparisons post-hoc test. The correlations were assessed with Pearson’s test. Finally, a receiver operating characteristic (ROC) curve analysis was used to describe the discriminatory accuracy of the lipoprotein parameters for subclinical atherosclerosis.

Statistical comparisons were considered significant at *p* < 0.05. All graphs and statistical analyses were obtained using GraphPad Prism version 9.0 for Windows (GraphPad Software, San Diego CA, USA).

## Results

### Severe Obesity Characteristics and Lipid Profile Assessed in Routine Clinical Practice

First, we analyzed the clinical characteristics of the cohorts and the results of the routine clinical assessment of the lipid parameters usually employed in evaluating the cardiovascular risk (Table 1). The mean BMI of the 37 subjects with SO was 47.5 kg/m^2^. The total cholesterol and LDL-C values obtained from the routine clinical analysis were in the desirable range according to the European Atherosclerosis Society (serum total cholesterol < 200 mg/dL and LDL-C below 116 mg/dL). In this population with SO, HDL-C was near the target levels (> 50 mg/dL in women and > 40 mg/dL in men), while TGs were at the recommended levels (< 150 mg/dL) [22].

**Table 1:** Clinical characteristics and routine clinical assessment of the lipid profile of the group with severe obesity

	Baseline	Follow-up after BS (*n* = 24)				
Routine clinical parameters	SO (*n* = 37)	With plaque (*n* = 7)		Without plaque (*n* = 17)		*p*-value
		SO	12 M	SO	12 M	Plaque/time
Sex (female/male)	26/11	4/3		14/3		ns
Age (years)	44	46		42		ns
BMI (kg/m^2^)	47.5 ± 5.9	45.1 ± 6.3	29.1 ± 5.1ººº	49.0 ± 6.6	28.9 ± 5.1ººº	ns/< 0.0001
DLP + (n (%))	14 (38%)	3 (43%)	0	2 (12%)	0	ns/ns
T2D + (n)	7 (19%)	2 (29%)	0	1 (6%)	0	ns/ns
TC (mg/dL)	185.0 ± 33.7	204.4 ± 39.1	169.9 ± 24.1º	172.8 ± 27.5	159.0 ± 30.6	ns/0.0028
LDL-C (mg/dL)	113.8 ± 29.2	117.1 ± 43.3	98.4 ± 18.5º	101.6 ± 28.6	93.1 ± 30.9	ns/0.0067
HDL-C (mg/dL)	46.0 ± 9.7	44.3 ± 8.2	54.1 ± 11.2	45.2 ± 7.3	52.4 ± 11.1º	ns/0.0027
TG (mg/dL)	141.1 ± 71.2	143.1 ± 46.0	83.7 ± 17.3ººº	130.8 ± 40.5	79.9 ± 23.9ººº	ns/< 0.0001

Parameters are expressed as the mean ± SD. Follow-up data were analyzed by two-way ANOVA and the Bonferroni post-hoc test or by a chi-square test for the presence of comorbidities. Abbreviations: SO, severe obesity; n, number of subjects; BMI, body mass index; DLP +, subjects with dyslipidemia; T2D +, subjects with type 2 diabetes mellitus; TC, total cholesterol; LDL-C, cholesterol in low-density lipoproteins; HDL-C, cholesterol in high-density lipoproteins; TG, triacylglyceride

Symbol (º) denotes differences *vs* SO, same group

One symbol, *p* < 0.05; three symbols, *p* < 0.001

Comparing the groups with and without plaques, we did not find differences in the BMI or age. Interestingly, routine clinical assessment of lipid parameters showed no significant differences between groups. However, in the overall population, a higher proportion of individuals with plaques had dyslipidemia (Table 2). Additionally, SCORE values did not differ between groups but it is important to note that this risk algorithm was not designed for populations with obesity and multiple comorbidities.

**Table 2:** Presence of comorbidities and CVR SCORE in baseline depending on plaque presence

		Baseline (*n* = 37)		
		With plaque (*n* = 13)	Without plaque (*n* = 24)	*p*-value
BMI (kg/m^2^)		45.0 ± 5.1	48.9 ± 5.9	ns
DLP + (%)		61.5	16.6	0.0097
T2D + (%)		38.4	8.33	ns
SCORE (%)	0—1%	66.6	87.5	ns
	2—3%	33.3	8.33	
	4%	0	4.16	

BMI is expressed as the mean ± SD. Differences between groups were analyzed by student t-test or by a chi-square test. Abbreviations: *n*, number of subjects; BMI, body mass index; DLP +, subjects with dyslipidemia; T2D +, subjects with type 2 diabetes mellitus

After BS, the atheromas did not show remission in the subjects with plaques, while subjects without plaques did not develop new ones. The BMI was reduced significantly in both groups (approximately 40% decrease). T2D and DLP were in remission in both groups. Total cholesterol, LDL-C, and TG levels significantly decreased after BS, whereas HDL-C levels increased.

### NMR Lipoprotein Profile in Severe Obesity

Once we measured the lipid profile obtained from routine clinical practice, we analyzed in depth the lipoprotein profile obtained with the NMR technique in our study population with SO (*n* = 37). Table 3 shows the NMR lipoprotein profile in the subjects with SO compared to the control group (32 women and 8 men, mean age of 43 years, healthy BMI of < 25 kg/m^2^). In general, we found altered lipoprotein parameters in SO. To note, NMR-determined total cholesterol, total TGs, LDL-C, and HDL-C (in Table 3) strongly correlated with the same parameters measured in clinical routine practice (shown in Table 1) (p < 0.0001 in all cases).

**Table 3:** Baseline NMR lipoprotein profile in the control group and the group with severe obesity

NMR PARAMETERS		Control (*n* = 40)	SO (*n* = 37)	*p*-value
VLDL	VLDL-C (mg/dL)	10.4 ± 5.5	14.8 ± 6.3	0.0007
	VLDL-TG (mg/dL)	44.4 ± 17.2	63.8 ± 24.8	< 0.0001
	VLDL-P number (nmol/L)	33.3 ± 13.2	47.1 ± 19.1	< 0.0001
	Large VLDL-P (nmol/L)	0.9 ± 0.4	1.3 ± 0.4	0.0011
	Medium VLDL-P (nmol/L)	2.9 ± 1.6	4.7 ± 1.5	< 0.0001
	Small VLDL-P (nmol/L)	29.3 ± 11.7	41.1 ± 17.4	0.0002
	VLDL diameter (nm)	41.9 ± 0.4	42.1 ± 0.2	ns
IDL	IDL-C (mg/dL)	7.0 ± 2.3	8.7 ± 2.8	0.0074
	IDL-TG (mg/dL)	8.4 ± 2.1	9.3 ± 2.2	ns
LDL	LDL-C (mg/dL)	106.7 ± 14.1	120.9 ± 24.8	0.0092
	LDL-TG (mg/dL)	10.8 ± 2.7	12.9 ± 3.8	0.0084
	LDL-P number (nmol/L)	1081.6 ± 130.9	1217.0 ± 249.4	0.0127
	Large LDL-P (nmol/L)	154.9 ± 21.5	180.0 ± 30.6	< 0.0001
	Medium LDL-P (nmol/L)	265.3 ± 77.8	315.8 ± 113.5	0.0672
	Small LDL-P (nmol/L)	661.3 ± 71.4	720.2 ± 136.4	0.0752
	LDL diameter (nm)	20.9 ± 0.3	21.0 ± 0.3	0.0262
HDL	HDL-C (mg/dL)	55.0 ± 10.8	45.6 ± 5.6	< 0.0001
	HDL-TG (mg/dL)	15.2 ± 3.5	9.7 ± 3.4	< 0.0001
	HDL-P number (µmol/L)	28.4 ± 4.5	22.3 ± 2.9	< 0.0001
	Large HDL-P (µmol/L)	0.2 ± 0.02	0.26 ± 0.03	< 0.0001
	Medium HDL-P (µmol/L)	9.3 ± 1.5	8.7 ± 0.9	ns
	Small HDL-P (µmol/L)	18.9 ± 3.5	13.4 ± 3.1	< 0.0001
	HDL diameter (nm)	8.3 ± 0.1	8.4 ± 0.1	< 0.0001

Values are shown as the mean ± SD. Statistical differences between the control group and the SO group were calculated using unpaired Student’s t-test. Abbreviations: SO, severe obesity; TG, triacylglyceride; C, cholesterol; VLDL, very-low-density lipoprotein; IDL, intermediate-density lipoprotein; LDL, low-density lipoprotein; HDL, high-density lipoprotein; P, particle number; NMR, nuclear magnetic resonance; ns, non-significant. Statistical comparisons were considered significant at *p* < 0.05

Regarding the proatherogenic lipoprotein content, the cholesterol and TG contents in VLDL and LDL were higher in the subjects with SO. VLDL-P and LDL-P were also greater in the subjects with SO regardless of whether they were large, medium or small. IDL-C content, but not TG content, was higher in the SO group than in the control group.

Concerning the anti-atherogenic particles, the HDL-C and TG contents were lower in the subjects with SO. The number of these particles was significantly decreased in the SO group compared to the control group, except for the medium ones.

### NMR Lipoprotein Profile in Subclinical Atherosclerosis and the Impact of Bariatric Surgery

After assessing changes in the lipoprotein profile in individuals with SO, we aimed to examine variations in the NMR lipoprotein profile depending on the presence of plaques and after BS. These analyses were performed in the 24 subjects who had completed the 12-month follow-up. Table 4 presents the composition and diameter of the lipoproteins in both groups.

**Table 4:** NMR-determined lipoprotein content and diameter in subjects with or without plaques before and after bariatric surgery

NMR parameters		Control (*n* = 40)	With plaque		Without plaque		ANOVA *(p-value)*	
			SO (*n* = 7)	12 M (*n* = 7)	SO (*n* = 17)	12 M (*n* = 17)	Plaque	Time
Content (mg/dL)	VLDL-C	10.4 ± 5.5	14.8 ± 3.8^c^	10.1 ± 2.1	15.8 ± 7.0^cc^	9.8 ± 3.7ºº	ns	0.0033
	VLDL-TG	44.4 ± 17.2	63.7 ± 15.6^c^	48.7 ± 8.3	68.4 ± 29.0^cc^	45.9 ± 12.0ºº	ns	0.0050
	IDL-C	7.0 ± 2.6	9.7 ± 3.0^c^	6.7 ± 1.5	8.5 ± 2.7	7.8 ± 3.1	ns	0.0435
	IDL-TG	8.4 ± 2.1	9.9 ± 1.9	8.1 ± 1.0	9.4 ± 2.5	8.5 ± 2.3	ns	ns
	LDL-C	106.7 ± 14.1	134.7 ± 30.9^ccc^	115.1 ± 13.3	112.4 ± 14.1*	113.6 ± 22.5	ns	ns
	LDL-TG	10.8 ± 2.7	14.1 ± 4.4^c^	9.6 ± 2.4	12.3 ± 3.3	11.5 ± 4.3*	ns	0.0097
	HDL-C	55.0 ± 10.8	45.3 ± 4.0^cc^	56.7 ± 8.6ºº	45.0 ± 5.1 ^ccc^	58.6 ± 6.4ººº	ns	< 0.0001
	HDL-TG	15.2 ± 3.5	8.8 ± 1.7 ^ccc^	11.1 ± 2.7^c^	10.3 ± 4.3 ^ccc^	12.4 ± 4.0^c^	ns	0.0223
Diameter (nm)	VLDL diameter	41.9 ± 0.4	42.1 ± 0.3	42.2 ± 0.3	42.1 ± 0.2	42.1 ± 0.3	ns	ns
	LDL diameter	20.9 ± 0.3	20.9 ± 0.2	21.0 ± 0.3º	21.0 ± 0.3	21.2 ± 0.2	ns	0.0441
	HDL diameter	8.3 ± 0.1	8.3 ± 0.1	8.3 ± 0.1	8.4 ± 0.1	8.4 ± 0.1	ns	ns

Values are shown as the mean ± SD. Statistical differences were assessed using two-way repeated measures ANOVA (with/without plaques and time of follow-up) and the Bonferroni multiple comparisons post-hoc test. Abbreviations: NMR, nuclear magnetic resonance; SO, severe obesity; 12 M, 12 months after bariatric surgery; TG, triacylglyceride; C, cholesterol; VLDL, very-low-density lipoprotein; LDL, low-density lipoprotein; HDL, high-density lipoprotein; ns, non-significant

Symbol (*) denotes differences vs group with plaque, same timepoint

Symbol (º) denotes differences vs SO, same group

Symbol (^c^) denotes differences vs control group

One symbol, *p* < 0.05; two symbols, *p* < 0.01; three symbols, *p* < 0.001

The NMR-determined **cholesterol content** was generally the same in the groups with and without plaques and normalized after BS. Specifically, VLDL-C decreased by 32% in the subjects with plaques and by 38% in those without plaques. IDL-C content also decreased after BS in both groups. LDL-C showed a trend towards a reduction in the subjects with plaques (15% decrease), but this did not reach statistical significance. It remained the same after BS in subjects without plaques, who had the same LDL-C concentration as the control group since baseline. In the case of HDL, the cholesterol content increased similarly in both groups with and without plaques (28% on average).

Similarly, the NMR-determined **content of TG** in the different particles did not differ between the groups. Moreover, after BS, the content of TG in VLDL and LDL decreased in both groups, becoming similar to control values. VLDL-TG decreased by about 28% in both groups, while LDL-TG decreased by 32% in the subjects with plaques and 7% in those without plaques. The TG content in IDL remained the same after BS. The HDL-TG content increased regardless of whether the subjects had plaques (26%) or not (19%), and normalized in both groups after BS.

**Fig. 1:**
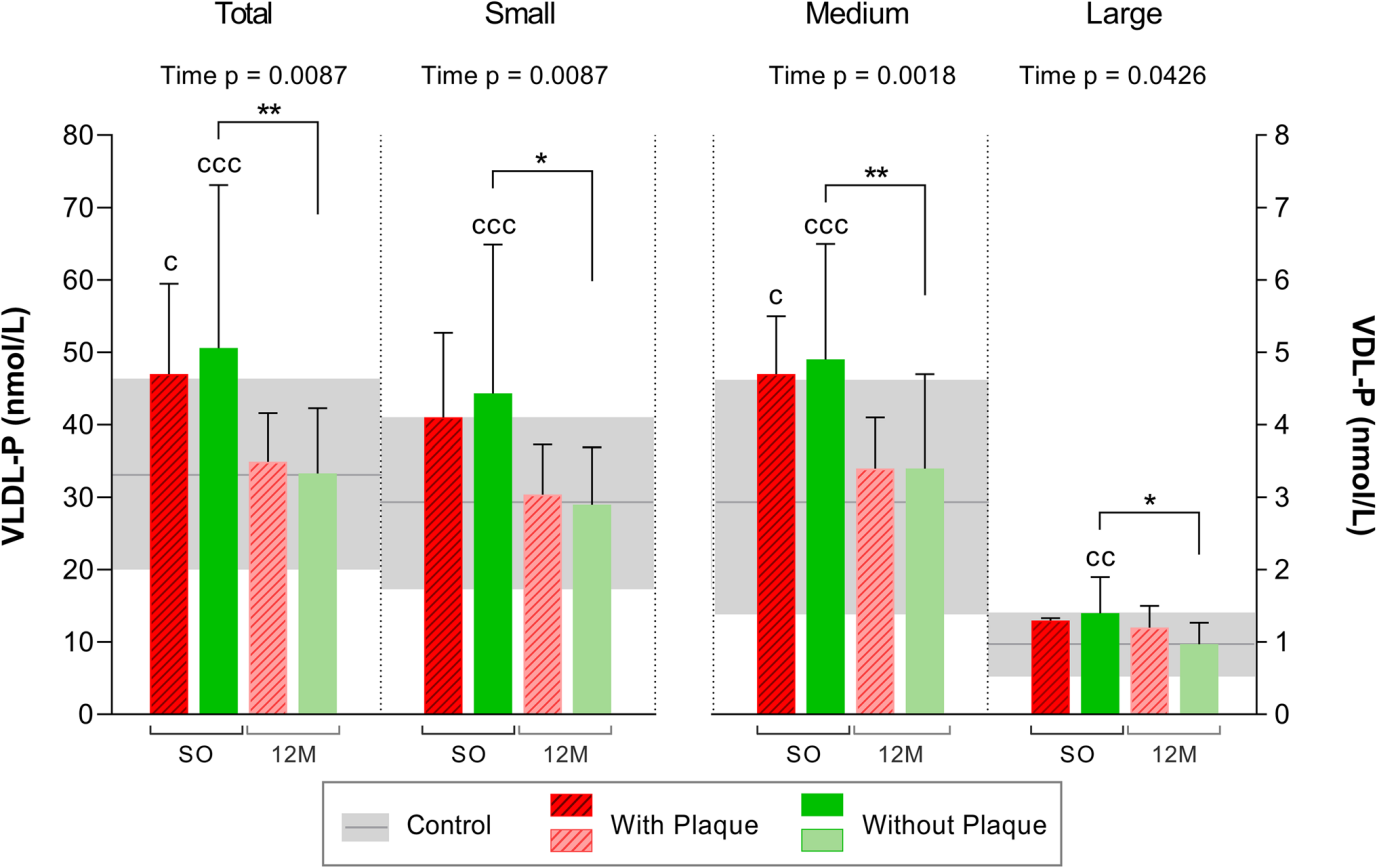
NMR-determined number of VLDL particles by size from subjects with or without plaques before and after bariatric surgery. *N* = 24. Values are shown as the mean, while the error bars show the SD. The horizontal gray bars represent the control group (mean ± SD). The left y-axis shows the total and small VLDL-P. The right y-axis shows the medium and large VLDL-P. Statistical differences were assessed using two-way repeated measures ANOVA (with/without plaques and time of follow-up) and the Bonferroni multiple comparisons post-hoc test. Abbreviations: SO, severe obesity; 12 M, 12 months after bariatric surgery; VLDL, very-low-density lipoprotein; P, particle number; NMR, nuclear magnetic resonance; ns, non-significant. Symbol (c) denotes differences vs control. Symbol (*) denotes differences vs other groups or time points. One symbol, p < 0.05; two symbols, p < 0.01; three symbols, p < 0.001

When focusing on particle number, the total **VLDL-P** value decreased significantly after BS, becoming equal to control levels (26% decrease in subjects with plaques and 34% in those without plaques) (Fig. 1). The most abundant VLDL particles were the smaller ones and the numbers of all subspecies also decreased after BS. The number of particles remained the same regardless of whether an atheroma was present or not throughout the study.

**Fig. 2:**
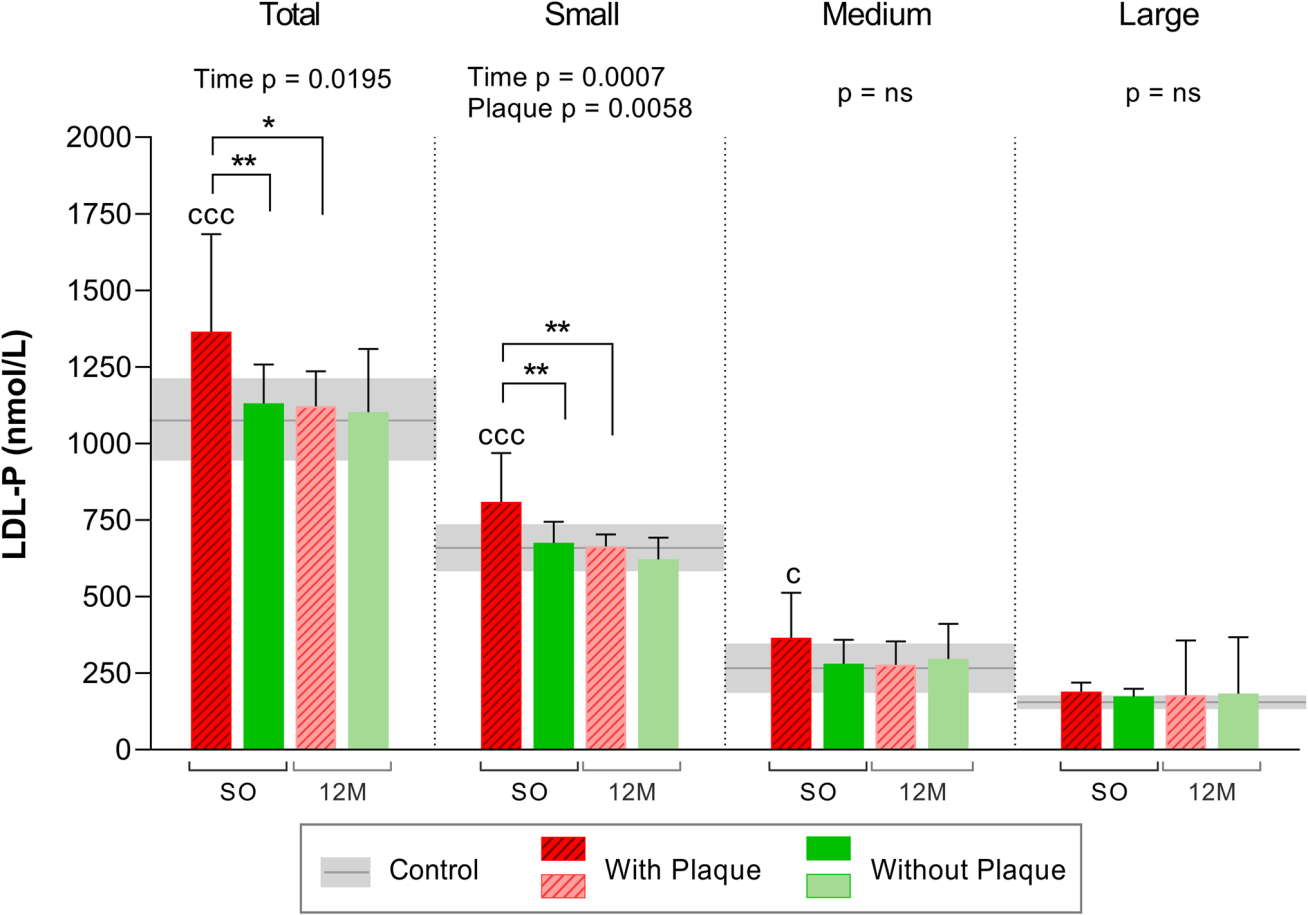
NMR-determined number of LDL particles by size in subjects with or without plaques before and after bariatric surgery. *N* = 24. Values are shown as the mean, while error bars show the SD. The horizontal gray bars correspond to the control group (mean ± SD). Statistical differences were assessed using two-way repeated measures ANOVA (with/without plaques and time of follow-up) and the Bonferroni multiple comparisons post-hoc test. Abbreviations: SO, severe obesity; 12 M, 12 months after bariatric surgery; LDL, low-density lipoprotein; P, particle number; NMR, nuclear magnetic resonance; ns, non-significant. Symbol (c) denotes differences vs control, symbol (*) denotes differences vs other groups or time points. One symbol, *p* < 0.05; two symbols, *p* < 0.01; three symbols, *p* < 0.001

Interestingly, in the subjects with SO before surgery, the total **LDL-P** value was higher in the group with plaques compared to the control group and the group without plaques (Fig. 2), but normalized after BS (18% decrease). Subjects without plaques had the same LDL-P value as controls all throughout the study. There were no differences between the subjects with and without plaques in the medium and large LDL-P. However, we found significant differences between the groups in the smallest and most abundant LDL particles, with the subjects presenting plaques showing a higher level of the small LDL-P (*p*_ANOVA_ = 0.0058). A clear improvement after BS was observed, as the value of the small LDL-P decreased at 12 M (*p* = 0.0007).

**Fig. 3:**
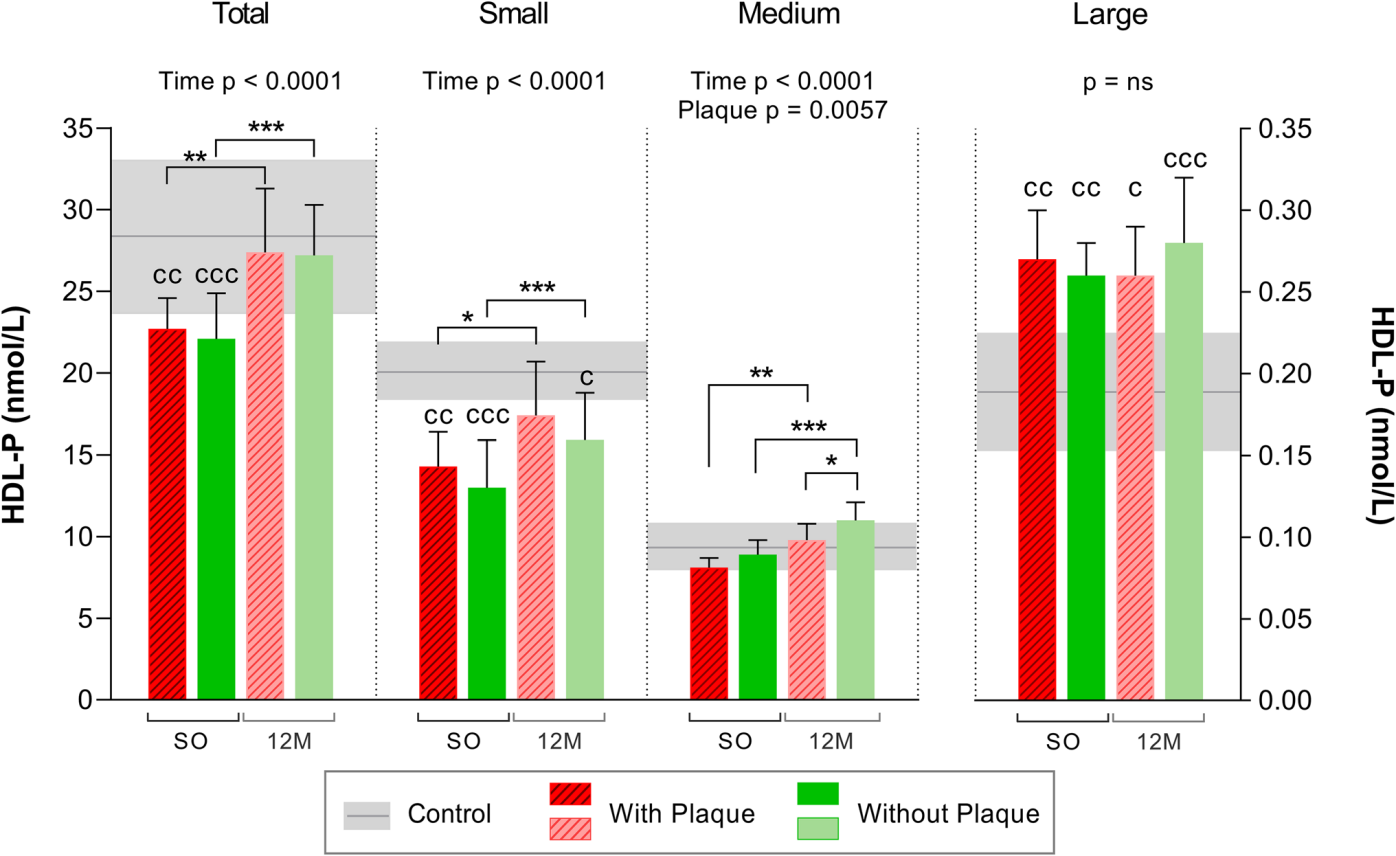
NMR-determined number of HDL particles by size in subjects with or without plaques before and after bariatric surgery. *N* = 24. Values are shown as the mean, while the error bars show the SD. The horizontal gray bars correspond to the control group (mean ± SD). The left y-axis shows the total, small, and medium HDL-P. The right y-axis shows the large HDL-P. Statistical differences were assessed using two-way repeated measures ANOVA (with/without plaques and time of follow-up) and the Bonferroni multiple comparisons post-hoc test. Abbreviations: SO, severe obesity; 12 M, 12 months after bariatric surgery; HDL, high-density lipoprotein; P, particle number; NMR, nuclear magnetic resonance; ns, non-significant. Symbol (c) denotes differences vs control, symbol (*) denotes differences vs other groups or time. One symbol, *p* < 0.05; two symbols, *p* < 0.01; three symbols, *p* < 0.001

The total **HDL-P** value normalized after BS in both groups (about 17% increase) (Fig. 3). After BS, both the small and the medium HDL-P values increased in the subjects with and without atheromas (*p*_ANOVA_ < 0.0001). Interestingly, the medium HDL-P value was higher in the subjects without plaques (*p*_ANOVA_ = 0.0057). The large HDL-P value remained higher in both groups than in the controls all throughout the study, but were the least prevalent among the HDL sizes.

### The Capability of Lipoproteins to Predict the Presence of Plaques in SO

Small LDL-P and medium HDL-P stood out as differential parameters between the subjects with plaques and those without plaques. Thus, we explored the ratio of small LDL-P to medium HDL-P as a preliminary approach to investigate whether it could provide an improved insight into the presence of atherosclerosis (Table 5). Interestingly, we found that this ratio was higher in the subjects with plaques.

**Table 5:** Ratio of small LDL-P to medium HDL-P determined by NMR in subjects with or without plaques before and after bariatric surgery

NMR parameters	Control (*n* = 40)	With plaque		Without plaque		ANOVA *(p-value)*	
		SO (*n* = 7)	12 M (*n* = 7)	SO (*n* = 17)	12 M (*n* = 17)	Plaque	Time
Small LDL-P/medium HDL-P	73.0 ± 15.1	99.5 ± 17.1^ccc^	68.8 ± 8.4 ººº	77.6 ± 14.1***	57.2 ± 9.4ººº^,cc^	< 0.001	< 0.001

Values are shown as the mean ± SD. Statistical differences were assessed using two-way repeated measures ANOVA (with/without plaques and time of follow-up) and the Bonferroni multiple comparisons post-hoc test. Abbreviations: NMR, nuclear magnetic resonance; SO, severe obesity; 12 M, 12 months after bariatric surgery; LDL, low-density lipoprotein; HDL, high-density lipoprotein; -P, particle number

Symbol (*) denotes differences vs group with plaques, same timepoint; symbol (º) denotes differences vs SO, same group; and symbol (^c^) denotes differences vs control group

Two symbols, *p* < 0.01; three symbols, *p* < 0.001

**Fig. 4:**
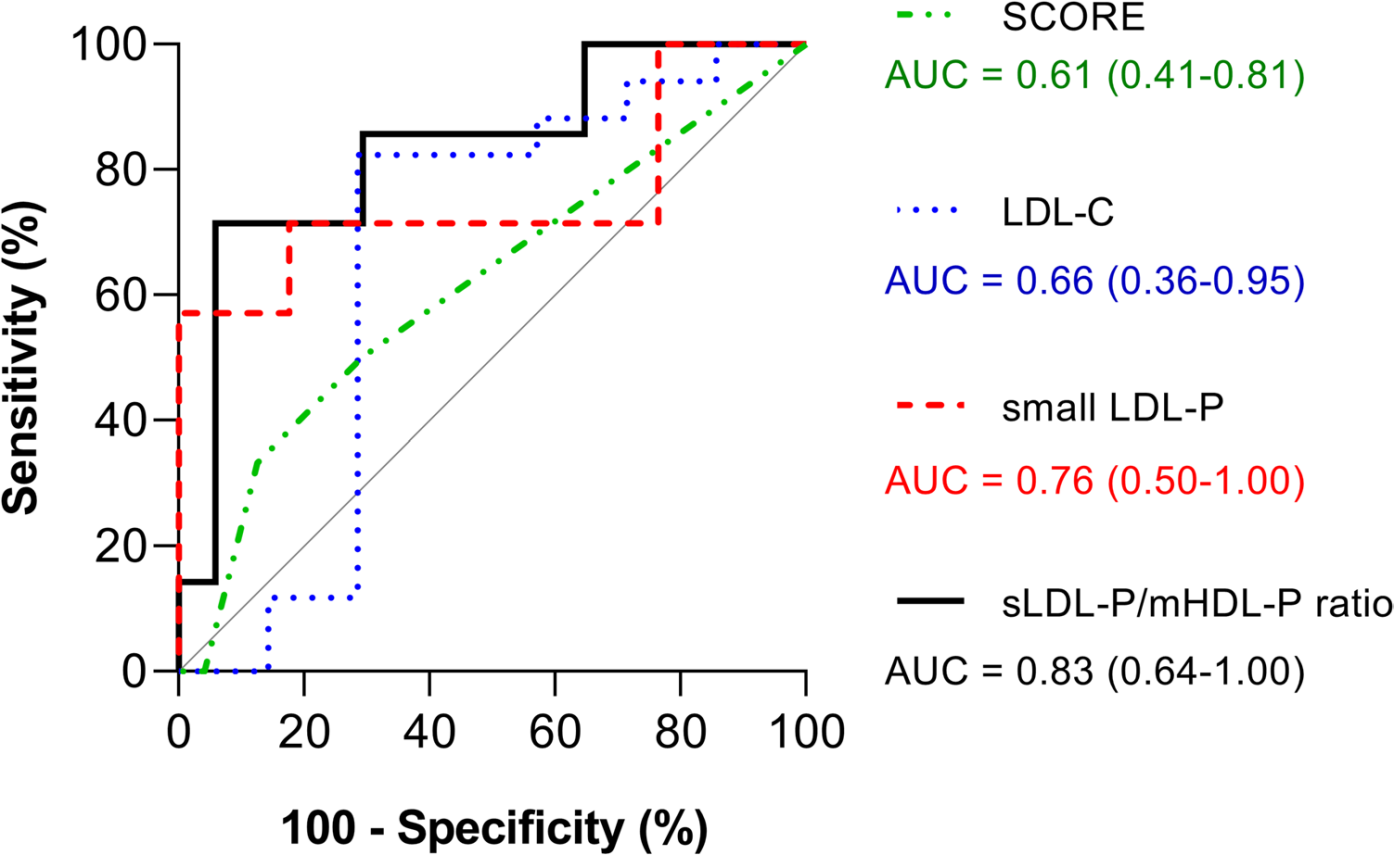
The prediction of the presence of plaques by SCORE, LDL-C, small LDL-P, and the ratio of small LDL-P/medium HDL-P in severe obesity. ROC curve analysis with the AUC and its 95% confidence intervals shown in brackets (*n* = 37 subjects). The diagonal gray line is the reference line (AUC = 0.5). Abbreviations: AUC, area under the curve; ROC, receiver operating characteristic; LDL-C, low-density lipoprotein cholesterol; LDL-P, number of particles of low-density lipoproteins; mHDL-P, particle number of medium high-density lipoproteins; SCORE, Systematic COronary Risk Evaluation; sLDL-P, particle number of small low-density lipoproteins

Figure 4 shows the ROC curves for the LDL-C (assessed in routine clinical practice) and the SCORE (recommended target and method for risk assessment and prevention in the recent guidelines of the European Society of Cardiology [23]), small LDL alone, and the calculated ratio. The area under the curve (AUC, indicating how much each parameter is capable of distinguishing between subjects with plaques and those without plaques) was higher for the calculated ratio, unveiling its better capability of predicting the presence of plaques. Furthermore, the narrower confidence interval indicates that the AUC value was more accurate for the ratio.

## Discussion

This study provides a comprehensive examination of advanced lipoprotein parameters in SO, their association with subclinical atherosclerosis, and the impact of BS. The present work unveiled proatherogenic lipoprotein abnormalities undetected by traditional methods that could be hallmarks of subclinical atherosclerosis in SO. In general, although the atheromas did not disappear, BS normalized the NMR-detected proatherogenic lipoprotein abnormalities found in SO. Interestingly, we found differences between subjects with plaques and those without in some of the particle subspecies, such as small LDL-P. Our study stands out as one of the few to report disparities in medium HDL-P. The ratio between these two parameters has potential in predicting subclinical atherosclerosis.

Regarding the pre-surgery routine clinical assessment of lipoprotein parameters (Table 1), it is interesting to observe that although the subjects with SO have a high cardiovascular risk, their total cholesterol, LDL-C, and TG levels were in the normal range [20]. Furthermore, these parameters were the same between subjects with plaques and those without. These results underscore the importance of characterizing a more advanced lipoprotein profile to elucidate the cardiovascular risk and its implications in subjects with SO.

Therefore, we determined the NMR lipoprotein profile to gain valuable insights into subclinical atherosclerosis in SO and to understand the influence of BS on the cardiovascular risk.

### NMR Lipoprotein Profile in SO and Subclinical Atherosclerosis

Concerning NMR-measured **VLDL**, the higher number of particles described in the 37 subjects in the SO group compared to the control group could be explained by the fact that excess adiposity leads to an increased release of free fatty acids by adipocytes [24]. This leads to an elevated uptake of free fatty acids in the liver, where they are used for TG synthesis. As a consequence, the synthesis and secretion of VLDL particles by the liver increase [24–26].

Following BS, the normalization of the total VLDL-P value in the 24 subjects with SO who had completed the follow-up reflects the decrease in the level of small VLDLs, the most atherogenic ones [27]. Furthermore, VLDL-TG content also decreased. A potential mechanism behind these decreases could be the increased insulin sensitivity after BS (insulin resistance in SO alters systemic lipid metabolism) [28, 29]. The changes in VLDL metabolism and TG content could be key contributors to the amelioration of the post-surgery cardiovascular risk.

VLDLs are used to derive LDL particles, which carry a large proportion of the cholesterol in circulation. As other studies have reported, we found a favorable reduction in the plasma concentration of NMR-detected LDL-C in subjects with plaques and SO undergoing BS [18, 30–33], but no differences were observed between the subjects with plaques and those without. On the contrary, we found differences in the number of total and small LDL-P between the subjects with plaques and those without. Hence, subjects with plaques had elevated levels of small LDL-P but similar LDL-C concentrations as the participants without plaques, indicating that the size of the LDL particles carrying the cholesterol could determine atherogenicity. The discrepancy between the concentration of LDL-C and the amount of proatherogenic LDL particles may result in misestimates of the cardiovascular risk when measuring LDL-C in SO [32].

An increasing amount of evidence is demonstrating that the small and medium LDLs are the most atherogenic particles, with the size of LDL being studied as a cardiovascular risk factor [34, 35]. Small LDL particles have a decreased affinity for the LDL receptor and a decreased clearance. They can more easily penetrate the arterial wall and undergo oxidation [36]. Interestingly, our previous study demonstrated increased levels of oxidized LDL in individuals with severe obesity and atheromatous plaques [37]. In the present study, we found a positive correlation between small LDL particles and oxidized LDL (r = 0.40, *p* = 0.0031), supporting this idea. After BS, we observed a decrease and normalization of the total LDL-P value, reflecting a lowering in the number of small particles despite the presence of plaques. Along this line, it is important to note the increase in the LDL diameter after BS. This could help to mitigate atheroma formation and may reflect a decrease in the cardiovascular risk [35].

NMR analysis directly quantifies **HDL** particle size, number, as well as cholesterol and TG contents [38]. Regarding HDL-C, the low levels observed before BS were likely to be caused by two main factors: an increase in TG-rich lipoproteins and the transfer of TGs into HDL particles [39–41]. The observed increase in TG-rich lipoproteins (as VLDL) stimulates the transfer of TGs to HDLs that is mediated by cholesteryl ester transfer protein (CETP). Consequently, HDL becomes TG-enriched and more prone to hydrolysis by hepatic lipase. This leads to the generation of smaller, rapidly metabolized HDL particles, resulting in reduced HDL-C levels. Both a decrease in the influx of lipoproteins from the diet and an increase in fatty acid turnover could explain the normalization of the HDL content after BS [42].

Although HDL plays a major role in reverse cholesterol transport and has beneficial biological properties against CVD [43], we observed in the present study that the total **HDL-P** was the same between subjects with plaques and those without. It has become apparent in recent years that in addition to HDL content and number, HDL size and functionality also play significantly important roles in atheroprotection [44, 45]. In this sense, our results show higher levels of medium HDL particles in subjects without plaques and an increase after BS.

A potential explanation for the increased concentration of medium HDL particles in individuals without plaques could be an increased activity of lecithin cholesterol acyl transferase (LCAT). LCAT plays a role in esterifying free cholesterol, transforming small HDL into medium ones. Alternatively, a reduced activity of CETP might contribute to the high medium HDL-P, as it converts medium HDL to large HDL by incorporating cholesterol esters [40].

The specific relationship of HDL particle size with the presence of an arterial plaque is still unknown and the research findings obtained to date can appear contradictory [44]. We suggest different factors that could explain the relationship between medium HDL and subclinical atherosclerosis. Medium HDL particles could be more effective at promoting the efflux of cholesterol from peripheral tissues, as they have more area than small ones and are more abundant than larger particles. On the contrary, small HDL particles have higher catabolic rates, contributing to an even lower HDL-C level [45].

Another explanation for the role of medium HDL in atherosclerosis is the protein paraoxonase 1 (PON1) that is associated with HDL. PON1 resides almost exclusively on HDLs and is critical for the capability of HDL to protect LDL and outer cell membranes against harmful oxidative modifications. Our previously published data showed that individuals with atheromatous plaques had significantly reduced PON1 levels (see reference [37] for detailed results) In accordance with this idea, in the present study, we found a significant positive correlation between PON1 levels and medium HDL-P (r = 0.57, *p* < 0.0001). Furthermore, a large proportion of PON1 might be carried by medium HDL, since they are more abundant than the large ones [38]. It is therefore possible that increased levels of medium HDL may be protective against subclinical atherosclerosis by carrying PON1 and effectively promoting the efflux of cholesterol [46–49].

### The Potential of Lipoproteins in the Detection of Subclinical Atherosclerosis in SO

It is well known that SO, related comorbidities, and subclinical atherosclerosis confer a high cardiovascular risk. Moreover, detecting plaques via ultrasound, if available, is especially challenging in subjects with SO and traditional lipid measurements are not precise for risk assessment in this population [3, 11–13].

In fact, managing a persistent cardiovascular risk in subjects with SO remains a clinical challenge [17, 50]. In this context, we conducted a detailed lipoprotein profile analysis using NMR, revealing characteristics not detectable by standard clinical laboratory techniques.

After the NMR analysis, we proceeded with an exploratory study to investigate whether specific characteristics of lipoproteins could be used as a complementary method for identifying the cardiovascular risk by predicting the presence of arterial plaques in SO.

We found two NMR-detected lipoprotein parameters that could be used to differentiate between subjects with plaques and those without: small LDL-P and medium HDL-P. It appears that these parameters play crucial roles in atherosclerosis. The decrease in medium HDL levels in subjects with plaques suggests reduced oxidative protection, while the observed increase in small LDL levels makes them more susceptible to penetrating the arterial wall and undergoing oxidation. Furthermore, the medium- and small-sized particles are proportionally abundant in the pattern of each lipoprotein. Therefore, we hypothesized that the ratio of small LDL-P to medium HDL-P may provide additional information about the cardiovascular risk and serve as a potential biomarker indicating the presence of a plaque. The obtained AUC for the ratio indicates a considerably high predictive capacity of 83%. Furthermore, this ratio is superior to small LDL-P alone, to the routine clinical assessment of LDL-C and also higher than SCORE in predicting subclinical atherosclerosis. Hence, the obtained ratio might provide valuable information about the presence of atheromas, which is crucial for assessing cardiovascular health.

Although this is a preliminary study with a small number of subjects, it provides a basis for future research on the detection of subclinical atherosclerosis. Furthermore, the low intra- and inter-assay variability as well as the reliability of the NMR method contribute to the robustness of the data despite the sample size.

## Conclusion

In the context of SO and its associated comorbidities, advanced NMR-detected parameters, such as lipoprotein number and size, provide more specific information on the proatherogenic lipid profile and its evolution after BS than the routine clinical assessment of lipid parameters.

Although further studies are needed to validate this proposal, we suggest that the ratio of small LDL-P to medium HDL-P has the potential for detecting and managing subclinical atherosclerosis in individuals with SO.

## Data Availability

The datasets generated during the current study are available from the corresponding author on reasonable request.

## Abbreviations

CVDs: Cardiovascular diseases
DLP: Dyslipidemia
SO: Severe obesity
TGs: Triacylglycerides
HDL-C: High-density lipoprotein cholesterol
LDL-C: Low-density lipoprotein cholesterol
NMR: Nuclear magnetic resonance
P: Particle number
VLDL: Very-low-density lipoprotein
IDL: Intermediate-density lipoprotein
BS: Bariatric surgery
T2D: Type 2 diabetes mellitus
BMI: Body mass index
HT: Hypertension
NCEP: National Cholesterol Education Program
ROC: Receiver operating characteristic
AUC: Area under the curve
CETP: Cholesteryl ester transfer protein
LCAT: Lecithin cholesterol acyl transferase
PON1: Paraoxonase 1
